# Supplementary material for: Energy Dense Salty Food Consumption Frequency Is Associated with Diastolic Hypertension in Spanish Children
Source: Nutrients. 2020 Apr 9;12(4):1027. doi: 10.3390/nu12041027 (PMC7230361; doi:10.3390/nu12041027)
Supplement: Supplementary file 1 [file nutrients-12-01027-s001.pdf]

SUPLEMENTARY MATERIALS

Table S1. General characteristics of the studied population.

	**Prepubertal**						**Pubertal**					
	**Normal weight**		**Overweight**		**Obesity**		**Normal weight**		**Overweight**		**Obesity**	
**N (%)**	**100 (29.7%)**		**64 (19%)**		173 (51.3%)		87 (25.2%)		101 (29.3%)		157 (45.5%)	
	**Mean**	**SD**	**Mean**	**SD**	**Mean**	**SD**	**Mean**	**SD**	**Mean**	**SD**	**Mean**	**SD**
Age	8.8[a]	1.8	9.7[b]	1.7	8.7[a]	1.8	12.5	1.6	12.3	1.8	12.3	1.6
Weight (kg)	27.6[a]	14.0	42.8[b]	10.5	49.1[c]	12.2	44.8[a]	10.2	57.6[b]	10	75.3[c]	16.2
Height (cm)	1.3[a]	0.1	1.4[b]	0.1	1.4[c]	0.1	1.6	0.1	1.5	0.1	1.6	0.1
WC (cm)	57.6[a]	14.0	74.6[b]	8.5	83[c]	10.1	67.1[a]	8.1	81.4[b]	9.2	97.3[c]	11.8
BMI z score	-0.4[a]	1.9	1.2[b]	0.6	3.2[c]	1.2	-0.2[a]	0.5	1.4[b]	0.5	3.2[c]	1.0
SBP (mmHg)	99[a]	11	106[b]	12	109[b]	13	104[a]	12	109[b]	13	117[c]	13
DBP (mmHg)	62[a]	9	63[a]	10	66[b]	10	62[a]	9	65[a]	10	70[b]	11
MAP (mmHg)	74[a]	7.7	77.2[a]	10.1	80.4[b]	8.8	75.9[a]	8.5	79.7[b]	8.7	85.6[c]	9.4
TAG (mg/dL)	52[a]	20	66[b]	32	73[b]	35	59[a]	24	73[b]	36	79[b]	34
CHOL (mg/dL)	173[a]	27	164[a]	34	165[b]	26	157	28	161	30	158	29
LDL-C (mg/dL)	95	25	94	29	98	24	85[a]	24	95[b]	27	94[b]	26
HDL-C (mg/dL)	67[a]	16	55[b]	14	49[c]	12	59[a]	13	49[b]	11	45[b]	12
Glucose (mg/dL)	84	7	84	6	83	9	86	8	87	9	85	7
Insulin (mU/l)	5.9[a]	3.7	9.4[b]	7.0	11.3[b]	7.18	10.3[a]	5	12.4[a]	7.1	18.9[b]	12.2
HOMA-IR	1.23[a]	0.82	1.99[b]	1.59	2.38[b]	1.59	2.19[a]	1.08	2.71[a]	1.62	4.01[b]	2.66

Different superscript letters indicate significant differences ($p < 0.05$) in the t-student test among children with normal weight, overweight and obesity in each prepubertal and pubertal stage. CHOL: Total Cholesterol; DBP: diastolic blood pressure; HDL-C: High density lipoprotein cholesterol; HOMA-IR: homeostasis model assessment for insulin resistance; LDL-C: Low density lipoprotein cholesterol; MAP: Mean arterial blood pressure (2*DBP+SBP)/3; SBP: systolic blood pressure; SD: Standard deviation; TAG: Triglycerides; WC: waist circumference.

Table S2. Types of food consumption and prepubertal stage in the three levels of systolic and diastolic blood pressure.

	Prepubertal														
	Normal BP				Elevated BP				HTN I and II				Linear General Model p-value		
	**N**	**Mean**	**SD**	**95%CI**	**N**	**Mean**	**SD**	**95%CI**	**N**	**Mean**	**SD**	**95%CI**	**P1**	**P2**	**P3**
**SBP**															
EDSF	225	13.3	8.8	(12.20–14.41)	24	13.8	10.1	(11.00–17.91)	60	11.8	7.6	(9.50–13.90)	0.191	0.231	0.319
HSF	173	21.5	12.6	(19.16–22.66)	20	20.4	12.9	(17.91–28.54)	45	19.1	9.2	(16.82–23.89)	0.549	0.687	0.662
SSB	223	8.4	16.3	(6.65–10.70)	24	14.9	18.0	(8.18–20.77)	59	7.0	9.8	(2.43–10.52)	0.173	0.171	0.108
MDASH	225	17.7	4.9	(17.24–18.48)	24	18.6	4.9	(15.66–19.51)	60	18.1	4.9	(16.30–18.76)	0.863	0.869	0.881
**DBP**															
EDSF	255	12.7	7.6	(11.64–13.69)	15	11.9	8.1	(8,08–16,82)	39	15.8	13.1	(13.32–18.66)	0.084	0.05	0.074
HSF	197	21.4	12.1	(19.61–22.86)	11	18.8	9.6	(11.60–26.05)	30	18.7	13.0	(15.84–24.32)	0.550	0.824	0.738
SSB	252	9.0	16.2	(7.18–10.97)	15	5.1	12.3	(-1.93–14.19)	39	7.8	11.1	(2.22–12.07)	0.669	0.645	0.632
MDASH	255	17.6	4.9	(17.14–18.29)	15	18.1	5.0	(15.16–20.06)	39	18.9	4.8	(16.72–19.71)	0.378	0.839	0.820

General linear model adjusted by center, sex, age, maternal education (P1), plus BMI (P2), plus physical activity (P3). BP: blood pressure; DBP: Diastolic blood pressure; EDSF: Energy dense salty food (times/week); HSF: High sugar foods (times/week) ; HTN: hypertension; MDASH: Modified dietary approach to stop hypertension (score); SBP: Systolic blood pressure; SD: Standard deviation; SSB: Sugar sweetened beverages(times/week) .

Table S3. Types of food consumption and pubertal stage in the three levels of systolic and diastolic blood pressure.

	Pubertal														
	Normal BP				Elevated BP				HTN I and II				Linear General Model p-value		
	**N**	**Mean**	**SD**	**95%CI**	**N**	**Mean**	**SD**	**95%CI**	**N**	**Mean**	**SD**	**95%CI**	**P1**	**P2**	**P3**
**SBP**															
EDSF	216	12.1	8.3	(11.11–13.31)	31	14.0	8.1	(10.89–16.74)	59	13.0	8.7	(10.54–14.98)	0.764	0.590	0.594
HSF	173	20.2	12.7	(17.92–21.72)	22	24.8	16.0	(17.97–28.87)	48	19.4	11.1	(16.65–24.39)	0.302	0.143	0.479
SSB	213	6.7	12.2	(5.00–8.83)	31	6.4	8.8	(1.92–12.01)	59	10.5	20.0	(5.99–13.64)	0.451	0.495	0.418
MDASH	216	18.5	4.9	(18.07–19.30)	31	17.6	4.8	(15.72–19.00)	59	19.6	5.1	(17.76–20.25)	0.118	0.104	0.252
**DBP**															
EDSF	262	12.2	8.1	(11.15–13.09)	12	11.6	6.3	(7.78–17.31)	32	15.3	10.5	(12.49–18.21)	0.157	0.101	0.116
HSF	207	20.3	12.8	(18.49–21.90)	9	21.6	21.2	(9.26–26.72)	27	21.2	8.4	(16.64–26.36)	0.885	0.644	0.767
SSB	259	6.7[a]	11.5	(4.98–8.33)	12	4.3[ab]	4.1	(-3.40–12.91)	32	14.1[b]	26.6	(10.10–19.89)	**0.006**	**0.006**	**0.006**
MDASH	262	18.7	4.8	(18.21–19.32)	12	18.6	5.2	(15.61–20.99)	32	18.3	6.2	(15.86–19.10)	0.648	0.180	0.333

Different superscript letters (a, b) indicate significant differences ($p<0.05$) among normal BP, risk of elevated BP and risk of hypertension I or II. T*he general linear model.* adjusted by center, sex, age, maternal education (P1), plus BMI (P2), plus physical activity (P3). BP: blood pressure; CI: Confidence interval; DBP: Diastolic blood pressure; EDSF: Energy dense salty food (times/week); HSF: High sugar foods (times/week); HTN: hypertension; MDASH: Modified dietary approach to stop hypertension (score); SBP: Systolic blood pressure; SD: Standard deviation; SSB: Sugar sweetened beverages(times/week) .

Table S4. Energy Dense Salty Foods (EDSF) consumption frequency in children with normal or excess-weight and the three diastolic blood pressure levels.

	Normotension				Elevated HTN				HTN I and II				Linear General Model p-value		
	**N**	**Mean**	**SD**	**95%CI**	**N**	**Mean**	**SD**	**95%CI**	**N**	**Mean**	**SD**	**95%CI**	**P1**	**P2**	**P3**
Normal weight	160	13.6[a]	7.5	(12.19–14.77)	7	15.3[a,b]	8.3	(8.01–20.54)	9	22.1[b]	18.2	(16.19–27.21)	**0.019**	**0.019**	**0.018**
Overweight	129	11.3	7.6	(10.00–12.85)	6	9.0	3.2	(0.71–14.10)	10	15.5	15.6	(9.78–21.13)	0.135	0.169	0.157
Obesity	228	12.4	8.1	(11.20–13.30)	15	11.4	7.3	(9.34–18.50)	52	14.4	10.2	(12.14–16.60)	0.276	0.198	0.212

Different superscript letters (a, b) indicate statistical significance of the general linear model adjusted by center, sex, age, maternal education (P1), plus BMI (P2), plus physical activity (P3). CI: Confidence interval; HTN: Hypertension; SD: Standard deviation.

Table S5. Food group consumption frequency by EDSF quartiles and pubertal stage.

	EDSF <P25th				EDSF P25th-P75th				EDSF >P75th				Linear General Model p-value		
	**N**	**Mean**	**SD**	**95%CI**	**N**	**Mean**	**SD**	**95%CI**	**N**	**Mean**	**SD**	**95%CI**	**P1**	**P2**	**P3**
**Prepubertal**															
HSF (times/week)	78	17.5a	11.2	(15.84–22.16)	175	20.2ab	10.9	(17.87–21.97)	84	25.0b	14.2	(21.40–26.98)	**0.027**	**0.031**	0.085
SSB (times/week)	78	8.4	14.4	(3.55–10.89)	175	8.4	16.1	(6.03–10.82)	84	10.8	17.6	(8.43–15.53)	0.159	0.161	0.069
MDASH (score)	78	19.5a	4.7	(18.04–20.19)	175	17.6ab	4.8	(16.86–18.28)	84	16.8b	5.1	(16.13–18.21)	**0.026**	**0.044**	0.119
**Pubertal**															
HSF (times/week)	90	16.5	11.7	(13.96–20.66)	171	20.0	13.3	(17.65–22.04)	84	23.4	12.5	(19.79–25.69)	0.067	0.065	0.052
SSB (times/week)	90	3.6a	9.3	(-0.62–5.51)	171	7.4b	14.1	(5.03–9.22)	84	12.8c	17.4	(11.22–17.61)	**0.000**	**0.000**	**0.000**
MDASH (score)	90	20.8a	5.2	(19.23–21.24)	171	18.8ab	4.6	(18.07–19.46)	84	16.4b	4.4	(16.18–18.29)	**0.001**	**0.000**	**0.001**

Statistical significance of the general linear model adjusted by center, sex, age, maternal education (P1), plus BMI (P2), plus physical activity (P3). EDSF: Energy dense salty food; HSF: High sugar foods; MDASH: Modified dietary approach to stop hypertension; SD: Standard deviation; SSB: Sugar sweetened beverages;.

Table S6. EDSF quartile consumption: anthropometric and metabolic variables divided by Tanner stage.

	Prepubertal												Pubertal											
	< P25th			P25th-P75th			>P75th			Linear General Model p-value			< P25th			P25th-P75th			>P75th			Linear General Model p-value		
	**N**	**Mean**	**SD**	**N**	**Mean**	**SD**	**N**	**Mean**	**SD**	**P1**	**P2**	**P3**	**N**	**Mean**	**SD**	**N**	**Mean**	**SD**	**N**	**Mean**	**SD**	**P1**	**P2**	**P3**
Weight (kg)	78	43.4	12.9	175	40.6	13.5	84	41.9	15.7	0.455	0.922	0.589	90	64.3	15.5	171	62.3	18.5	84	60.7	19.3	0.807	0.291	0.430
WC (cm)	78	77.5	11.9	77.5	175	13.9	84	72.9	15.6	0.156	0.206	0.150	90	86.6	13.6	171	84.9	16.7	84	83.3	16.4	0.728	0.166	0.189
MAP (mmHg)	78	79.2	10.4	175	76.9	8.6	84	78.5	8.7	0.197	0.301	0.378	90	80.4	10.6	171	81.2	9.4	84	82.9	9.7	0.178	0.132	0.098
SBP (mmHg)	78	107	13.7	175	105	11.8	84	104	13.1	0.392	0.607	0.515	90	111	14.2	171	111	14.5	84	114	12.6	0.086	0.059	**0.048**
DBP (mmHg)	78	65	10.3	175	63	9.4	84	65	9.4	0.140	0.178	0.258	90	65	12.1	171	66	9.8	84	67	10.3	0.441	0.416	0.337
TAG (mg/dL)	78	67	36.3	175	64	31.3	84	66	29.7	0.800	0.940	0.960	90	66	27.7	171	75	37.0	84	74	33.1	0.270	0.250	0.279
CHOL (mg/dL)	78	167	34.7	175	165	23.8	84	172	28.5	0.139	0.137	0.081	90	161	33.9	171	159	27.5	84	155	27.5	0.576	0.578	0.509
LDL (mg/dL)	78	100	30.4	175	93	22.9	84	100	22.7	0.114	0.119	0.081	90	93	30.7	171	94	24.5	84	87	22.8	0.486	0.471	0.462
HDL (mg/dL)	78	50	12.4	175	56	15.0	84	58	17.5	0.142	0.209	0.256	90	49	12.2	171	49	13.5	84	53	12.6	0.264	0.201	0.226
Glucose (mg/dL)	78	83	9.1	175	84	7.1	84	84	7.3	0.929	0.941	0.984	90	85	8.5	171	86	7.8	84	87	7.1	0.776	0.778	0.812
Insulin (mU/l)	78	9.9	6.9	175	9.3	6.7	84	8.6	6.6	0.341	0.377	0.247	90	13.1	7.3	171	15.3	9.1	84	15.7	13.9	0.460	0.355	0.545
HOMA	78	2.1	1.6	175	2.0	1.5	84	1.8	1.4	0.379	0.390	0.315	90	2.8	1.7	171	3.3	2.0	84	3.4	3.0	0.602	0.491	0.674

General linear model adjusted by center, sex, age, maternal education (P1), plus BMI (P2), plus physical activity (P3). CHOL: Total Cholesterol; DBP: diastolic blood pressure; HDL-C: High density lipoprotein cholesterol; HOMA-IR: homeostasis model assessment for insulin resistance; LDL-C: Low density lipoprotein cholesterol; MAP: Mean arterial blood pressure (2*DBP+SBP)/3; SBP: systolic blood pressure; SD: Standard deviation; TAG: Triglycerides; WC: waist circumference.
